# YCZ-18 Is a New Brassinosteroid Biosynthesis Inhibitor

**DOI:** 10.1371/journal.pone.0120812

**Published:** 2015-03-20

**Authors:** Keimei Oh, Tadashi Matsumoto, Ayumi Yamagami, Atushi Ogawa, Kazuhiro Yamada, Ryuichiro Suzuki, Takayuki Sawada, Shozo Fujioka, Yuko Yoshizawa, Takeshi Nakano

**Affiliations:** 1 Department of Biotechnology, Faculty of Bioresource Sciences, Akita Prefectural University, Shimoshinjo Nakano, Akita, Japan; 2 Antibiotics laboratory, RIKEN, 2–1 Hirosawa, Wako, Saitama, Japan; 3 Department of Bioproduction, Faculty of Bioresource Sciences, Akita Prefectural University, Shimoshinjo Nakano, Akita, Japan; 4 Biotechnology Research Center, Faculty of Bioresource Sciences, Akita Prefectural University, Shimoshinjo Nakano, Akita, Japan; 5 RIKEN Center for Sustainable Resource Science, Wako, Saitama, Japan; 6 CREST, Japan Science and Technology Agency, Kawaguchi, Saitama, Japan; Instituto de Biología Molecular y Celular de Plantas, SPAIN

## Abstract

Plant hormone brassinosteroids (BRs) are a group of polyhydroxylated steroids that play critical roles in regulating broad aspects of plant growth and development. The structural diversity of BRs is generated by the action of several groups of P450s. Brassinazole is a specific inhibitor of C-22 hydroxylase (CYP90B1) in BR biosynthesis, and the application use of brassinazole has emerged as an effective way of complementing BR-deficient mutants to elucidate the functions of BRs. In this article, we report a new triazole-type BR biosynthesis inhibitor, **YCZ-18**. Quantitative analysis the endogenous levels of BRs in *Arabidopsis* indicated that **YCZ-18** significantly decreased the BR contents in plant tissues. Assessment of the binding affinity of **YCZ-18**to purified recombinant CYP90D1 indicated that **YCZ-18** induced a typical type II binding spectrum with a K_d_ value of approximately 0.79 μM. Analysis of the mechanisms underlying the dwarf phenotype associated with **YCZ-18** treatment of *Arabidopsis* indicated that the chemically induced dwarf phenotype was caused by a failure of cell elongation. Moreover, dissecting the effect of **YCZ-18** on the induction or down regulation of genes responsive to BRs indicated that **YCZ-18** regulated the expression of genes responsible for BRs deficiency in *Arabidopsis*. These findings indicate that **YCZ-18** is a potent BR biosynthesis inhibitor and has a new target site, C23-hydroxylation in BR biosynthesis. Application of **YCZ-18** will be a good starting point for further elucidation of the detailed mechanism of BR biosynthesis and its regulation.

## Introduction

The oxidative metabolism of campesterol in plant tissues leads to the production of a group of bioactive polyhydroxylated steroids, collectively called brassinosteroids (BRs). BRs are important plant hormones that play critical roles in regulating broad aspects of plant growth and development [1]. BRs act as essential regulators in cell elongation, cell division, and sex determination [2–3]. Mutants with impaired BR synthesis display dramatic growth defects, such as decreased cell elongation, resulting in pleiotropic dwarf phenotypes, delayed flowering, and male sterility [4–7]. BRs also modulate plant metabolic pathways in response to environmental biotic and abiotic stress resistance, including tolerance of salt, drought and oxidative stresses and pathogen resistance [8, 9].

Because BRs are involved in controlling plant architecture, seed yields and stress resistance, manipulating BR levels in plant tissues is considered useful for enhancing crop production. The use of transgenic techniques to manipulate endogenous BR levels has a remarkable effect on plant growth. Overexpression of DWARF4, an enzyme that catalyzes a rate-limiting step in BR biosynthesis, enhances plant growth and seed yield in *Arabidopsis thaliana* (hereafter *Arabidopsis*) [10]. Similarly, transgenic rice plants overexpressing a sterol C-22 hydroxylase that catalyzes a key step in BR biosynthesis show increased biomass and seed yields [11], and available evidence indicates that mutations in BR biosynthesis may be a means to improve biomass production [12]. Consequently, the biosynthetic pathway of BRs is a potential target for engineering in terms of crop protection [13].

An alternative method for manipulating the BR levels in plant tissues is the use of specific inhibitors targeting the enzymes involved in BR biosynthesis. Because agrochemicals have been widely used for crop protection in the modern agricultural industry, this method has advantages over the use of BR-deficient mutants, as it can be used at different stages of plant growth and development [14]. Moreover, inhibitors can easily be applied to different plant species. In this context, the search for potent inhibitors of BR biosynthesis represents a worthwhile approach to develop new technologies for manipulating BR levels in plant tissues.

**Fig 1:**
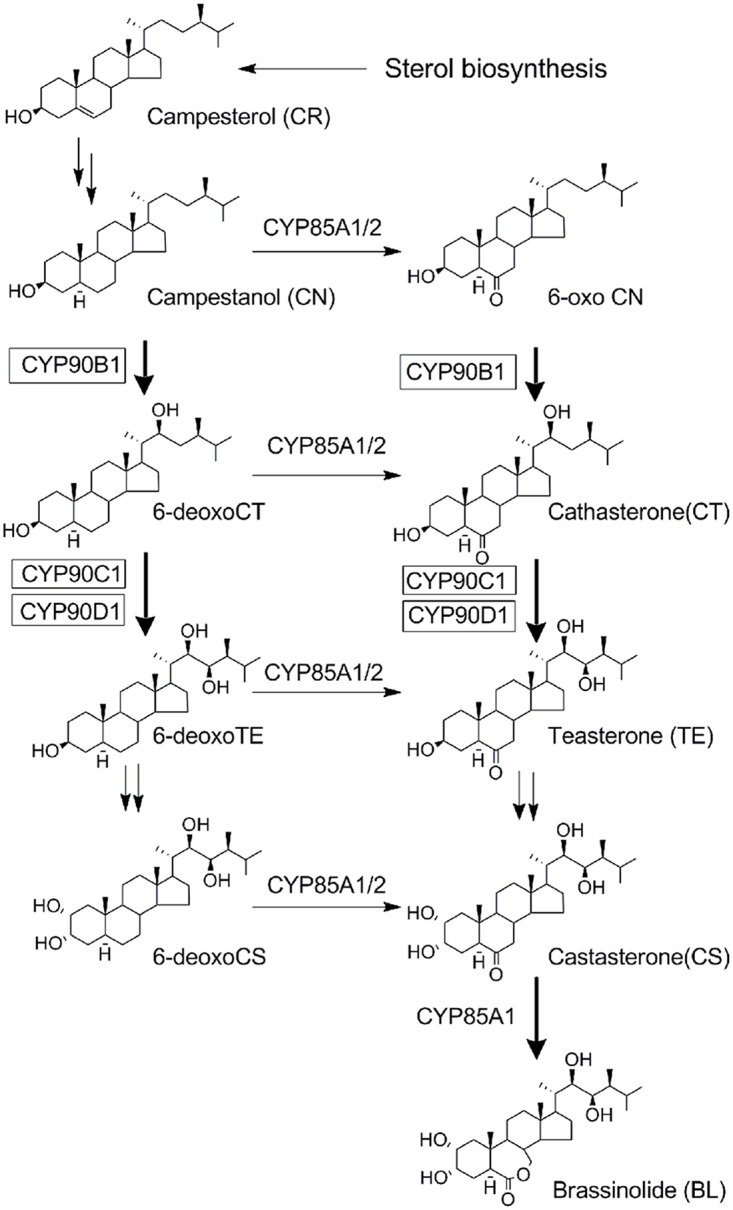
Outline of the BR biosynthetic pathway and P450.

The biosynthetic pathways of BRs were initially elucidated by tracer experiments using various labeled precursors of brassinolide (BL) in periwinkle (*Catharanthus roseus*) cell lines [15]. The pathways were later validated by analyzing the endogenous levels of BRs in BR-deficient mutants [4, 16]. With the combination of genetic and biochemical approaches, the entire metabolic pathway in the biosynthesis of BRs has been identified (Fig. 1) [17]. Molecular and functional analysis of the BR biosynthesis mutants demonstrated that BR biosynthesis was mediated by several cytochrome P450 monooxygenases (P450s). DWF4/CYP90B1 is thought to catalyze the C-22 hydroxylation of campesterol (CR) [16]. CPD/CYP90A1 is thought to be involved in the C-3 dehydrogenation of steroid skeletons [18]. CYP90C1/ROT3 and CYP90D1, which are genetically closely related, are shown to have redundant functions as C-23 hydroxylases [19]. *Arabidopsis* CYP85A1 and CYP85A2 were found to catalyze the C-6 oxidation reaction [20]. These observations indicate that many steps in BR biosynthesis are catalyzed by P450 enzymes (Fig. 1). Therefore, it is reasonable to postulate that the biosynthetic pathway of BRs is an expedient target for P450 inhibitors. Likewise, the multiplicity of P450s in BR biosynthesis suggests the possibility of developing P450 inhibitors targeting different steps of BR biosynthesis, thereby allowing us to probe the detailed mechanism of BR biosynthesis and its regulation.

The mechanisms of inhibition of P450 have been studied in considerable detail [21]. Triazole derivatives have been demonstrated to have widespread ability as P450 inhibitors due to the intrinsic affinity of the nitrogen electron pair in heterocyclic molecules for the prosthetic heme iron. The triazole derivatives thus bind not only to lipophilic regions of the protein but also simultaneously to the prosthetic heme iron [22]. Accordingly, the chemical structures beyond the triazole moiety in P450 inhibitors of the triazole type are of significant importance regarding the selectivity of P450 inhibition.

**Fig 2:**
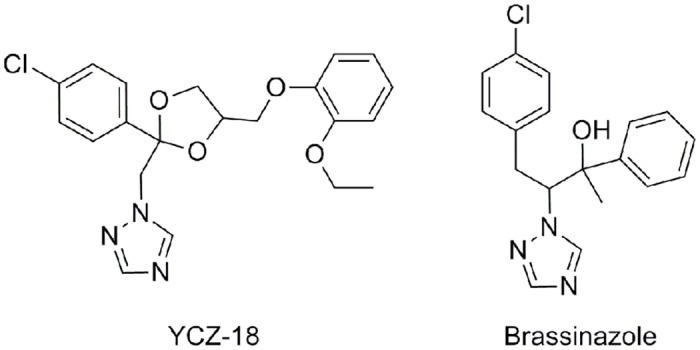
Chemical structures of brassinosteroid biosynthesis inhibitors.

Asami reported the discovery of brassinazole (chemical structure shown in Fig. 2), the first class of synthetic triazole-type inhibitors of BR biosynthesis [23–25]. Studies on the modes of action of brassinazole have shown that the target site of brassinazole is DWF4 (CYP90B1) [26]. To explore new inhibitors with novel target sites in BR biosynthesis, we have been developing P450 inhibitors targeting BR biosynthesis [27–30]. Using an approach based on ketoconazole as a molecular scaffold, we found a new series of inhibitors of BR biosynthesis **(YCZ-series)** [27]. Structure-activity relationship studies of **YCZs** revealed a highly selective and potent inhibitor: **YCZ-18** (chemical structure shown in Fig. 2) [28, 29]. Stereochemical structure-activity relationship studies led to the identification of **2*R*,4*S*-YCZ-2013** and **2*S*,4*R*-YCZ-2013**, analogues of **YCZ-18**, which are the most potent BR biosynthesis inhibitors found to date, with IC_50_ values of approximately 24 ± 2 and 24 ± 1 nM, respectively [30].

In the present work, we report a biochemical and physiological characterization of **YCZ-18**. Our results indicate that **YCZ-18** is a specific BR biosynthesis inhibitor with a wide range of applicability for altering BR biosynthesis in *Arabidopsis*. Assessment of the target site of **YCZ-18** indicates that one of its targets is CYP90D1, which differentiates **YCZ-18** from brassinazole, a known BR biosynthesis inhibitor.

## Materials and Methods

### Chemicals

Synthesis and purification of **YCZ-18** were carried out according to a method described previously [27]. Teasterone (TE) and brassinolide (BL) were purchased from Brassino Co., Ltd. (Toyama, Japan). Cathasterone (CT) was provided by Professor Suguru Takatsuto of Joetsu University of Education. Brz 220 was a gift provided by Professor Tadao Asami of The University of Tokyo. Other reagents of the highest purity were purchased from Tokyo Chemical Industry Co., LTD. (Tokyo, Japan) or Wako Pure Chemical Industries, Ltd. (Tokyo, Japan). All the chemicals for biological studies, unless otherwise described, were dissolved in DMSO and stored at -30°C before use.

### Plant materials and growth conditions

*Arabidopsis thaliana* Columbia-0 (Col-0) was used in all the experiments. BR-deficient mutant *de-etiolation2* (*det2*) [31] and Brz220, a potent BR biosynthesis inhibitor [23], were used as positive controls. Untreated plants were used as controls for comparison with the chemical-induced phenotype. Plants were germinated and grown on half-strength Murashige and Skoog (MS) medium containing 1.0% sucrose and 0.9% phyto-agar (Duchefa), in the presence or absence of inhibitors and/or BRs. Seed sterilization and seed handling were carried out as described previously [29]. Conditions in the growth chamber were 16 h light (100 μE m^-2^ sec^-1^ white light)/8 h dark at 22°C unless otherwise indicated. For gene expression analysis, ground tissue from whole seven-day-old seedlings was used.

### YCZ-18 treatment under soil and hydroponic culture conditions

*Soil*: Seeds were sown in soil and grown in controlled environments (16/8 h light/dark cycle, 22°C, 50–60% relative humidity). Ten-day-old *Arabidopsis* plants were sprayed with an aqueous solution of **YCZ-18** (5 μM containing 0.1% DMSO, approximately 0.1 pmol/plant). Two days later, **YCZ-18** was sprayed on plants in the same way for the second time, and the plants were allowed to continue to grow in soil for observations of chemical-induced phenotypes. Plants sprayed with an aqueous solution of DMSO were used as a control.

*Hydroponics*: Sterilized seeds were sown on sponges (approximately 0.2 cm thick) saturated with half MS solution in 0.9% phyto-agar. The seeds were placed in a hydroponic culture system that was prepared as previously described [32]. Twenty-day-old plants were placed and grown in culture medium [32] with the indicated concentrations of **YCZ-18** (0.1, 0.5, 1 μM). Plants grown in culture medium without **YCZ-18** were used as controls.

### Quantitative analysis of brassinosteroids

To determine the endogenous levels of BRs, seedlings of *Arabidopsis* (wild type (control), YCZ-18-treated (3μM), Brz-treated (3μM)) were harvested to yield approximately 15 g fresh weight tissues. The tissues were extracted twice with 250 ml of methanol. Deuterium-labeled internal standards (1.5 ng/g fresh weight) were added to the extracts. Purification and quantification of brassinosteroids were performed according to the methods described previously [33, 34].

### Microscopic analysis

Plants treated without or with **YCZ-18** (1 μM) were grown under hydroponic conditions in a growth chamber under standard conditions, as described above. The inflorescence stems from seven-week-old plants were dissected. The tissue was fixed in FAA for 16 h at 4°C, dehydrated with a graded ethanol series, embedded in paraffin, sectioned longitudinally in 10-μm slices using a microtome (Microm HM360, Microm, Laborgerät GmbH, Walldorf, Germany) and dried at 37°C. Paraffin was removed from the sections with xylene. The prepared slides were hydrated in an ethanol-water series and stained with hematoxylin. They were then dehydrated in a graded water-ethanol series, ethanol-xylene and then xylene. The slides were mounted with Eukitt and image data was captured under a microscope (BX51, Olympus, Japan). The cell length of pith in stem was calculated from the image data using Image J (Version 1.48, National Institute of Health, USA). The length of ten cells with three replications was measured in each treatments and the mean and standard deviation were calculated.

### Quantitative real-time PCR

The methods for total RNA isolation, cDNA synthesis, and real-time PCR have been previously described [35]. The sequences of the gene-specific primers for real-time PCR were as follows: for *TCH4*, 5’- CGAGTCTTTGGAACGCTGAT-3’ and 5’-CTTCTTGTTGAAAGCCACGG-3’; for *DWF4*, 5’-CATAAAGCTCTCTTCAGTCACGA-3’ and 5’-CGTCTGTTCTTTGTTTCCTAA-3’; for *LHCP*, 5’-ATCCGACCGAGTCAAGTACT-3’ and 5’-GGTTCCTTGCGAATGTCT-3’; and for *rbcS*, 5’-GCACCGACTCCGCTCA-3’ and 5’-TGGACTTGACGGGTGTTGTC-3’.

### Construction of CYP90D1 expression vectors

*Arabidopsis* full-length cDNA was provided by the RIKEN BRC through the National Bio-Resource Project of the MEXT, Japan [36, 37]. The expression vector, pCold-GST, was obtained from Dr. C. Kojima of Osaka University [38]. The DNA fragment encoding CYP90D1 mature protein was generated by PCR with forward primer 5’-AATCGAGCTCATGGACACTTCTTCTTCACTTTTG-3’ and reverse primer 5’- TTGACTGCAGTTATATTCTTTTGATCCAAATGGGT-3’. The PCR product was digested with *Sac*I-*Pst*I and was inserted into the pCold-GST expression vector. All of the constructed plasmids were transferred to the BL21 star (DE3) strain of *E*. *coli* (Invitrogen). The transformed cell was incubated in 10 ml of Luria broth containing 100 μl/ml of chloramphenicol overnight at 37°C. The 10 ml of pre-culture was incubated in 1000 ml of Luria broth containing 100 μl/ml of ampicillin at 37°C.

### Expression and purification of recombinant CYP90D1

The expression and purification of recombinant CYP90D1 were performed as described previously [39]. The purified CYP90D1 was dialyzed for 6 h at 4°C by using an oscillatory dialysis system (Daiichi Pure Chemicals, Co. Ltd. Tokyo, Japan) against 2x300 ml dialysis buffer (50 mM sodium phosphate buffer, pH 7.0). Cleavage of the fusion protein was carried out by using HRV3C protease according to the supplier’s protocol (Takara, Bio., KK. Japan). Protein measurements were performed using a Protein Assay Kit (Bio-Rad, Hercules, CA, USA), using bovine serum albumin as a standard. The relative purity of recombinant CYP90D1 was estimated by SDS–polyacrylamide gel electrophoresis (12% polyacrylamide) and staining of gels with Coomassie Brilliant Blue R250.

### Binding assay of YCZ-18 to recombinant CYP90D1

Binding of **YCZ-18** to CYP90D1 was measured by optical difference spectroscopy of purified recombinant CYP90D1 using a Shimadzu UV3100 spectrophotometer. Purified recombinant CYP90D1 was diluted in 50 mM sodium phosphate buffer (pH 7.0) with 0.1% Tween 20 to a final concentration of 3.5 μM containing 20% glycerol and separated into two matched black-walled quartz cuvettes (500 μl). After establishing a baseline, 0.7 μl of **YCZ-18** (0.5 mM dissolved in Me_2_SO) was added to the sample cuvette. Equal volumes of Me_2_SO were added to the reference cuvette. The samples were allowed to equilibrate for 2 min, and the difference spectrum was determined between 370 and 500 nm. The final volume of additions was kept to <1% of the total volume. Changes in absorbance as a function of **YCZ-18** concentration (0.7, 1, 2, 4, 8, 12, 16 μM) were determined at wavelengths selected on the basis of the spectral characteristics of each individual sample. Data obtained were used to calculate binding constants based on linear regression analysis. Spectral determinations were performed at least twice for each experiment, confirming the reproducibility with respect to the spectral profile and the position of λmax and λmin.

### Statistical analysis

All measurements were carried out at least in triplicate. Data analysis (t-test and analysis of variance) was applied to determine the significant difference with the use of significance throughout the manuscript being based upon *P*<0.05 unless stated otherwise.

## Results

### Biological activities of YCZ-18 in Arabidopsis under different growth conditions

To elucidate the effects of **YCZ-18** on the growth of *Arabidopsis*, three complementary growth conditions were assigned to ensure the capture of data describing the impact of **YCZ-18** to *Arabidopsis* growth and development over the entire life of the plants. The first growth condition, growth on plates for a period of 1–2 weeks, demonstrates the effects of **YCZ-18** on early seedling growth. The second condition is a hydroponic growth condition that has been validated for studies on root development. The third condition consists of spraying the chemicals on the plants grown in soil for a period of approximately 2 months.

**Fig 3:**
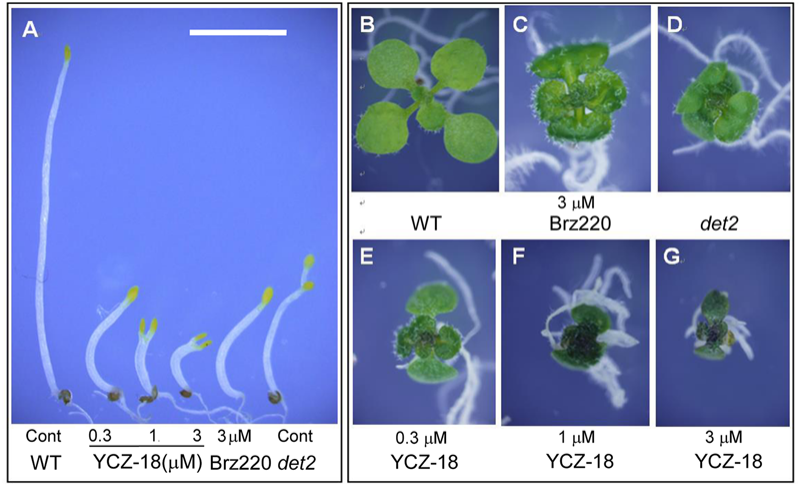
YCZ-18-treated plants display the BR-deficient phenotype. **YCZ-18**-treated plants (0.3, 1, 3 μM), Brz220-treated plants (3 μM) and brassinosteroid-deficient mutant (*det2*) plants were grown for 6 days in the dark (A) and for 10 days in the light (B-G) on medium containing the chemical indicated. The control plants (Cont) were untreated. Scale bar = 5 mm.

Fig. 3 shows the effects of **YCZ-18** on *Arabidopsis* grown on plates. We treated wild-type *Arabidopsis* plants with **YCZ-18** at concentrations ranging from 0.3 to 3 μM for 5 days after their germination on half MS agar-solidified medium under light (Fig. 3B-G) and dark (Fig. 3A) conditions. BR-deficient mutant *deetiolation2* (*det2*) [31] and wild-type *Arabidopsis* treated with Brz220 (3 μM) [23] were used as positive controls for comparison with the experimental phenotype. The hypocotyl of *Arabidopsis* seedlings without chemical treatment elongated to approximately 18 mm with closed cotyledons in the dark condition (Fig. 3A). In contrast, the *Arabidopsis* wild-type plants in **YCZ-18**-containing medium exhibited short hypocotyls and opened cotyledons, similar to the features of *det2* mutant and wild-type plants treated with Brz220 (Fig. 3A). The hypocotyl elongation was suppressed by **YCZ-18** in a dose-dependent manner. To compare the overall efficacy of **YCZ-18** with Brz220, we treated the plants with equal concentrations of inhibitors. Wild-type *Arabidopsis* plants treated with 3 μM **YCZ-18** displayed an approximately 90% reduction of hypocotyl length (Fig. 3A, third plant from the right), whereas those treated with Brz220 (3 μM) showed an approximately 80% reduction in hypocotyl length (Fig. 3A, second plant from the right). The degree of suppression of the hypocotyl elongation for Brz220 (3 μM) was similar to that treated with 0.3 μM **YCZ-18** (Fig. 3A, the second plant from the left). In the light condition, **YCZ-18** significantly reduced the rosette diameter (the plant size) of wild-type plants of *Arabidopsis* (Fig. 3E-G), to no larger than half the size of control plants (Fig. 3B). At a concentration of 0.3 μM **YCZ-18** (Fig. 3E), **YCZ-18** reduced plant size to a greater degree than *det2* (Fig. 3D) or 3 μM Brz220 treatment (Fig. 3C). The plants grown on the half MS agar-solidified medium containing **YCZ-18** showed dark green leaves, a common characteristic of BR-deficient mutants and Brz220-treated plants [23, 31].

**Fig 4:**
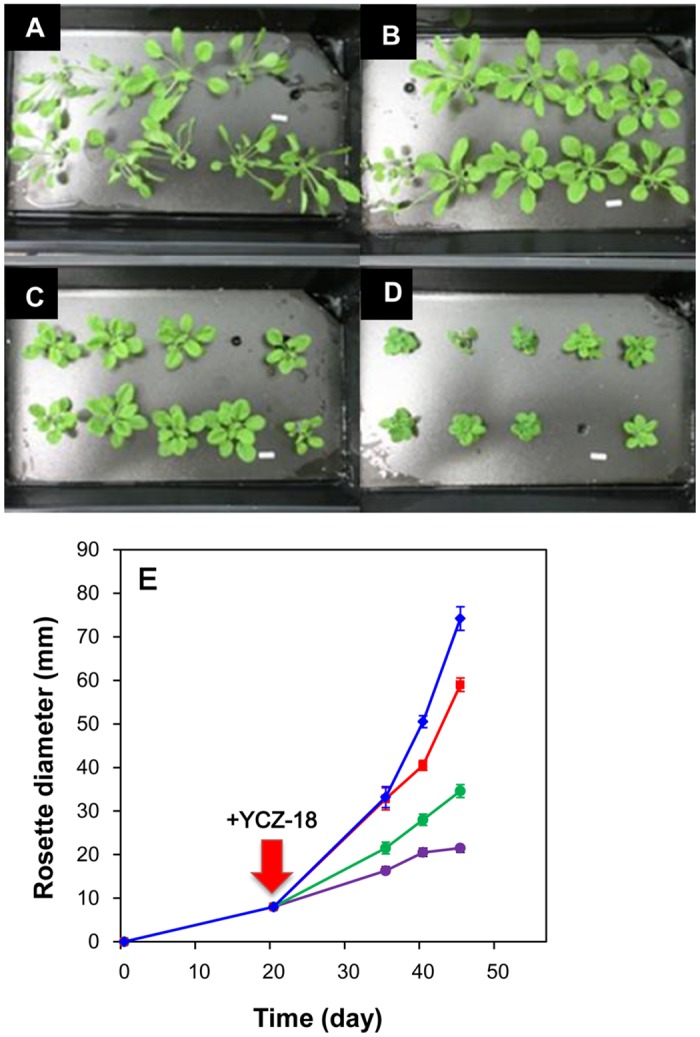
Effect of YCA-18 on the hydroponic growth of *Arabidopsis*. *Arabidopsis* plants grown under hydroponic conditions with or without **YCZ-18** treatment were treated as indicated in the methods section. Forty-five-day-old *Arabidopsis* (A); forty-five-day-old *Arabidopsis* treated with **YCZ-18** at 0.1 μM (B), 0.5 μM (C), or 1 μM (D). The growth curves of *Arabidopsis* treated with different concentrations of **YCZ-18** (E). Data are the means ± s.e. obtained from 8 to 10 plants. Bar = 1 cm.

In some cases, studies of plant root development are challenging. To evaluate the effect of **YCZ-18** on the growth of *Arabidopsis* under hydroponic conditions, a hydroponic system developed by Tocquin P. et al. was used in the present work [32]. We treated the twenty-day-old plants with **YCZ-18** at final concentrations of 0.1, 0.5 and 1 μM in the culture medium. The rosette diameter of the plants was used as a factor to evaluate the chemically induced dwarfism of *Arabidopsis*. In untreated plants, the rosette diameter increased as a function of time (Fig. 4E, blue diamond). In the presence of **YCZ-18**, the growth rate decreased with the increasing concentrations of **YCZ-18** (Fig. 4E). The rosette diameters for forty-five-day-old *Arabidopsis* without **YCZ-18** treatment were approximately 7.3±0.5 cm (Fig. 4A). In the presence of **YCZ-18** (0.1 μM), the rosette diameters were approximately 5.9±0.2 cm (Fig. 4B). The rosette diameters of plants in the presence of **YCZ-18** at final concentrations of 0.5 and 1 μM were found to be approximately 3.5±0.2 (Fig. 4C) and 2.1±0.2 cm (Fig. 4D), respectively. These results indicate that **YCZ-18** induces dwarfism of *Arabidopsis* under hydroponic conditions (*p*<0.05).

**Fig 5:**
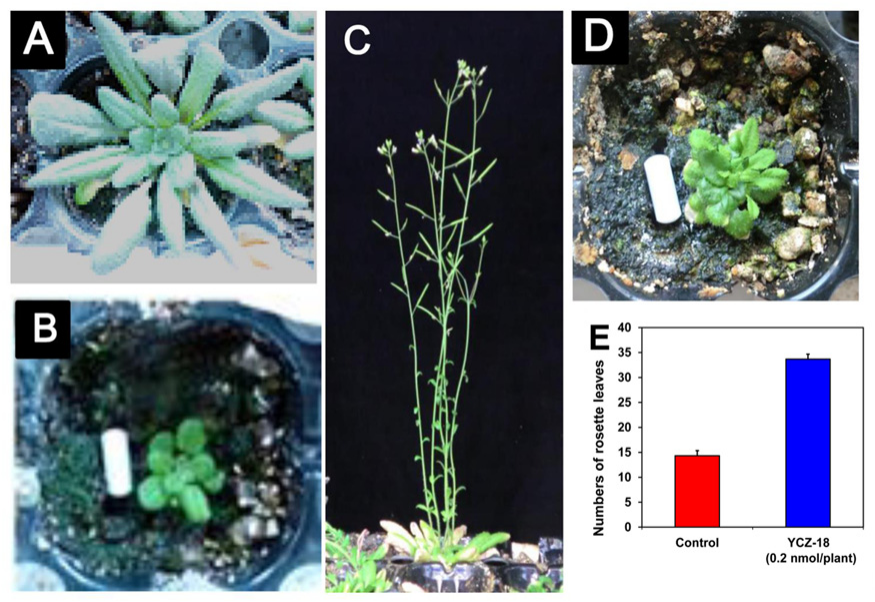
Effect of YCZ-18 on the growth of Arabidopsis in soil. The application of **YCZ-18** on wild-type *Arabidopsis* was performed by spraying an aqueous solution of **YCZ-18** (5 μM) onto ten-day-old wild-type *Arabidopsis* plants (approximately 0.2 pmol/plant), as indicated in the methods section. Four-week-old *Arabidopsis* seedlings (A), four-week-old *Arabidopsis* treated with **YCZ-18** (B), six-week-old *Arabidopsis* (C), six-week-old *Arabidopsis* seedlings treated with **YCZ-18** (D), rosette leaf number of six-week-old *Arabidopsis* at bolting from three plants (E). Data are the means ± s.e. obtained from 3 plants. Scale bar = 1 cm.

To further evaluate the biological activities of **YCZ-18** in *Arabidopsis*, we next examined the effects of **YCZ-18** on *Arabidopsis* grown in soil. The application of **YCZ-18** on wild-type *Arabidopsis* was conducted by spraying an aqueous solution of **YCZ-18** (5 μM) on the ten-day-old wild-type *Arabidopsis* (approximately 0.2 pmol/plant), as described in the methods section. The rosette diameter of four-week-old *Arabidopsis* treated with **YCZ-18** was approximately 1.2±0.1 cm (Fig. 5B), which was smaller than that of the control (approximately 8.5±0.5 cm, Fig. 5A). This result suggests that **YCZ-18** significantly induced dwarfism in *Arabidopsis*. After the plants had grown in soil for another 2 weeks, the plants without **YCZ-18** treatment grew to the reproductive stage (Fig. 5C), whereas the **YCZ-18**-treated plants remained in the vegetative stage (Fig. 5D). The number of rosette leaves of **YCZ-18-**treated plants averaged approximately 33.6 ±1.0 (leaves), whereas the control plants had 14.3±1.0 leaves (Fig. 5E). This result indicates that **YCZ-18** prolonged the vegetative stage in *Arabidopsis*.

### YCZ-18 reduces endogenous BR levels different from Brz.

To further investigate the action mechanism of **YCZ-18** on BR biosynthesis, we determined the endogenous levels of BRs in **YCZ-18**-treated *Arabidopsis*. Using gas chromatography-mass spectrometry (GC-MS) analysis together with deuterium-labeled BRs as internal standards [33, 34], we found that **YCZ-18** significantly decreased the endogenous BR levels in *Arabidopsis* (Table 1). In non-treated plants, the amount of castasterone, which is the adjacent precursor of brassinolide, was found approximately 0.19 ng/g fw, while the amount of castasterone was observed approximately 0.03 ng/g fw in **YCZ-18**-treated *Arabidopsis* (Table 1). This result indicated that **YCZ-18** significantly reduced the endogenous BR levels in *Arabidopsis*. Interestingly, the pattern of the levels of individual BR in **YCZ-18**-treated *Arabidopsis* was quite different from that of positive control Brz-treated *Arabidopsis*. As shown in Table 1, Brz decreased all the detectable BR at a level below 0.1 ng/g fw, but **YCZ-18** did not. The amount of 3-dehydro-6-deoxoteasterone, for example, was observed slightly increased in **YCZ-18**-treated *Arabidopsis* by the comparison with non-treated control (0.47 versus 0.35 ng/g fw.) (Table 1). This result indicates that **YCZ-18** has a different mode of action from Brz.

**Table 1:** Endogenous BR levels of control, YCZ-18 and Brz-treated Arabidopsis (ng/g fw).

	Control		YCZ-18 (3 μM)		Brz (3 μM)	
6-DeoxoBRs	1^st^ exp.	2^nd^exp.	1^st^ exp.	2^nd^exp.	1^st^ exp.	2^nd^exp.
6-Deoxocathasterone	1.83	1.99	0.19	0.27	0.05	0.07
6-Deoxoteasterone	0.05	0.11	0.07	0.08	nd*	nd
3-Dehydro-6-deoxoteasterone	0.30	0.40	0.53	0.41	0.02	0.05
6-Deoxotyphasterol	1.53	1.78	0.92	0.98	0.01	0.02
6-Deoxocastasterone	2.87	3.04	0.18	0.23	0.06	0.07
6-OxoBRs						
Cathasterone	nd	nd	nd	nd	nd	nd
Teasterone	nd	nd	nd	nd	nd	nd
3-Dehydroteasterone	nd	nd	nd	nd	nd	nd
Typhasterol	0.09	0.10	nd	0.02	nd	nd
Castasterone	0.15	0.22	0.01	0.05	0.03	0.02
Brassinolide	nd	nd	nd	nd	nd	nd

*nd: not detected (below detection limit)

### YCZ-18 induced dwarfism of Arabidopsis owing to a reduction in cell length

**Fig 6:**
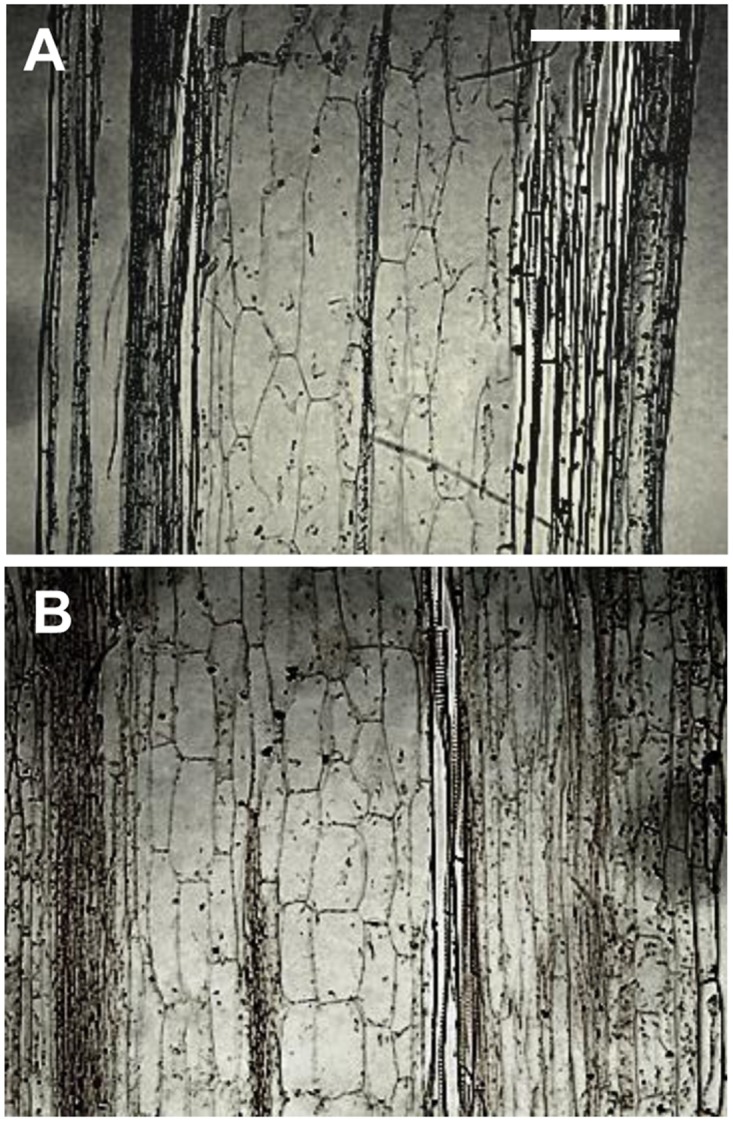
Longitudinal sections of YCZ-18 treated and untreated plant tissues. Stem from a seven-week-old control plant grown under hydroponic conditions. Average cell length of pith is 203.4 ± 12.5 μm (A). Stem from a seven-week-old **YCZ-18**-treated (1 μM) plant. Average cell length is 76.0 ± 1.0 μm (B). *Arabidopsis* plants were grown as shown in Fig. 4(A) And (B) are at the same magnification; Bar = 150 μm.

The short stature and the reduction in growth rate of **YCZ-18-**treated *Arabidopsis* could be due to a reduction in cell expansion or elongation. To gain insight into the mechanisms underlying the morphological changes associated with **YCZ-18** treatment, we performed a histological analysis of inflorescence stems of *Arabidopsis* grown under hydroponic conditions. Our transverse stem sections indicate that the dwarfism of the morphological phenotypes is driven by a significant decrease in cell elongation (Fig. 6A, 6B). The average cell length of pith in **YCZ-18**-treated (1 μM) plants was statistically (*p*<0.001) shorter than that of the control plants (76.0 ± 0.6 versus 203.4 ± 7.2 μm). Therefore, the short stature and reduced tissue/organ size seen in **YCZ-18**-treated plants are largely or exclusively due to a failure of individual cell elongation.

### YCZ-18 regulated the expression of BR-responsive genes

**Fig 7:**
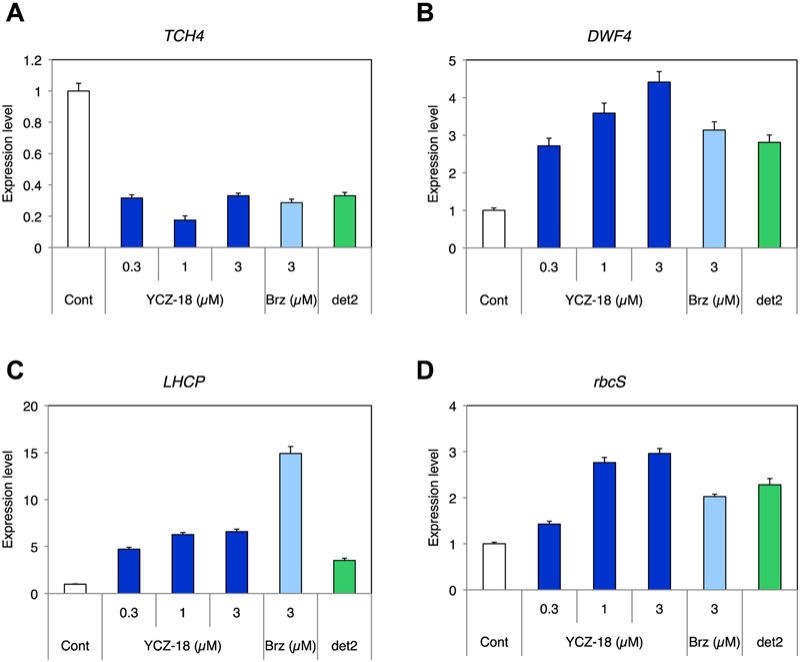
YCZ-18 regulates the expression of BR-responsive genes. Quantitative RT-PCR experiment measuring the relative expression levels of a BR-upregulated gene (*TCH4*) (A), a BR biosynthetic gene (*DWF4*) (B) and two photosynthesis genes (*LHCP* (C) and *rbcS* (D)) of the wild-type plant (Cont), **YCZ-18**-treated (0.3, 1, 3 μM), Brz-treated (3 μM) and brassinosteroid-deficient mutant *det2*. Plants were grown for 10 days in the light (A, B) and for 6 days in the dark (C, D) on a medium containing the chemical indicated. All results are means ± s.e.

To further investigate the mechanism of **YCZ-18** action in *Arabidopsis* at the molecular level, we assessed the effects of **YCZ-18** on BR-responsive gene expression. Seven-day-old wild-type plants of *Arabidopsis* grown on half MS agar-solidified media treated with or without **YCZ-18** were used for quantitative real-time PCR (qPCR) analysis. The concentrations of **YCZ-18** used were 0.3, 1, and 3 μM, concentrations at which **YCZ-18** promisingly induces BR-deficiency-like phenotypes in *Arabidopsis* (Fig. 2). As shown in Fig. 7, the BR-positive regulatory gene *THC4*, which encodes xyloglucan endotransglycosylase [40], showed lower expression, and the BR biosynthetic gene *DWF4* encoding CYP90B1, which is downregulated with BR stimulation through a feedback mechanism [41], showed higher expression in wild-type *Arabidopsis* grown on **YCZ-18** than in plants grown on the control medium without **YCZ-18** in the light (Fig. 7A, B).

BR-deficient mutant *det2* and wild-type plants treated with brassinazole showed increased expression of photosynthesis genes in the dark, which is a phenomenon known as ‘de-etiolation in the dark by BR-deficiency [25, 31, 42]. In general, the photosynthesis genes, *rbcS*, encoding the small subunit of ribulose 1,5-diphosphate carboxylase [43], and *LHCP*, encoding light-harvesting chlorophyll a/b-binding proteins [44], are used as genetic markers for light and BR-deficiency responses [25, 31]. As shown in Fig. 7C and 7D, under dark conditions, the expression levels of these two photosynthesis genes were higher in wild-type *Arabidopsis* treated with **YCZ-18** than those without **YCZ-18** treatment. In addition, the positive control of Brz220-treated wild-type *Arabidopsis* and the BR-deficient mutant *det2* display similar patterns in the up- and downregulation of the expression of these genes (Fig. 7).

### YCZ-induced short hypocotyls were rescued by the brassinosteroid biosynthesis intermediate teasterone but not by cathasterone

**Fig 8:**
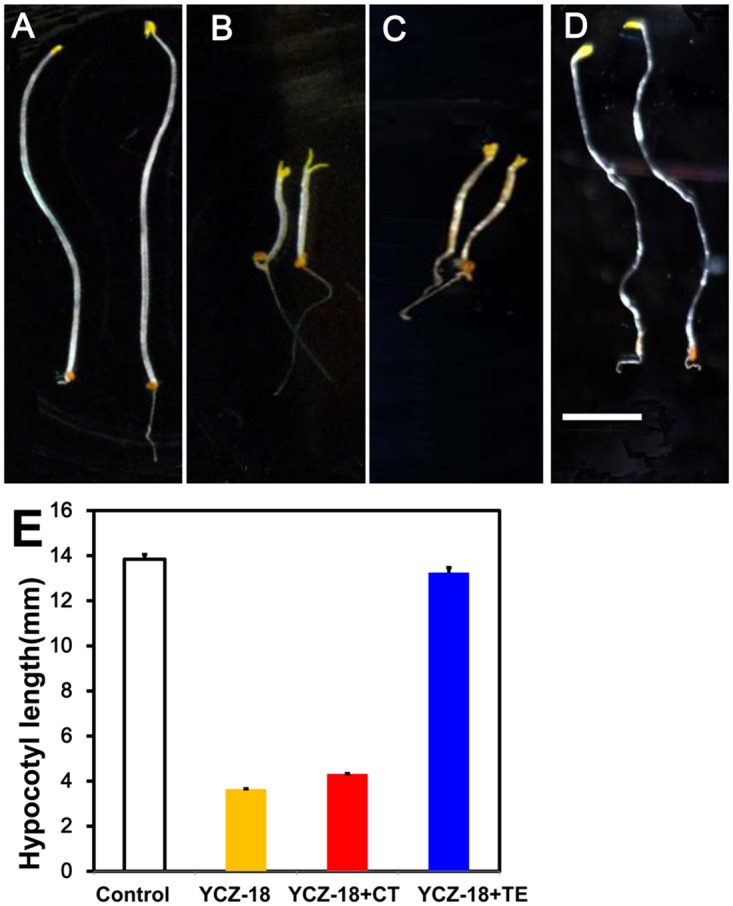
YCZ18-treated Arabidopsis in response to cathasterone (CT) and teasterone (TE). Five-day-old *Arabidopsis* seedlings (8A, 8E, white bar), treated with 0.5 μM **YCZ-18** (8B, 8E, yellow bar), treated with 0.5 μM **YCZ-18** together with 30 μM cathasterone (CT) (8C, 8E, red bar), or treated with 0.5 μM **YCZ-18** together with 10 μM teasterone (TE) (8D, 8E, blue bar). Data are the means ± s.e. obtained from 30 seedlings. Scale bar = 3 mm.

We have previously shown that **YCZ-18**-induced short hypocotyls of dark-grown *Arabidopsis* seedlings can be restored by the application of teasterone (TE) but not by campestanol (CN) (Fig. 1) [29]. With this observation, the candidate target site of **YCZ-18** in BR biosynthesis has been narrowed down to the following two steps. One step is the C-22 hydroxylation of CN (or 6-oxoCN) to 6-deoxocathasterone (6-deoxoCT) (or CT), which is catalyzed by CYP90B1 (DWF4) [16]. The second step is the C-23 hydroxylation of 6-deoxoCT (or CT) to 6-deoxoTE (or TE), which is catalyzed by CYP90C1/ROT3 and/or CYP90D1, two closely related enzymes with redundant functions (Fig. 1) [19]. To identify the target site of **YCZ-18** between these two steps, we conducted a stepwise feeding experiment by using CT and TE to test whether CT could rescue the **YCZ**-induced dwarfism of *Arabidopsis* seedlings grown in the dark. As shown in Fig. 8, in the presence of 0.5 μM **YCZ-18**, the hypocotyl length of *Arabidopsis* seedlings was approximately 2.6±0.4 mm, whereas the hypocotyl length of the controls was approximately 13.8±0.5 mm. When TE (10 μM) was added to the growth medium, the hypocotyl length of *Arabidopsis* seedlings was restored from 3.6±0.3 to 13.2±0.5 mm. This result indicates that TE reversed the **YCZ-18**-induced dwarfism of *Arabidopsis* seedlings grown in the dark, implying that the enzymes downstream of TE were not inhibited by **YCZ-18**. Application of CT (30 μM) did not reverse the **YCZ-18**-induced dwarfism as the hypocotyl length shifted from 3.6±0.3 to 4.2±0.2 mm, suggesting that **YCZ-18** inhibited enzymes downstream of CT. Taking these results together with our previous observations [29], the target site of **YCZ-18** in BR biosynthesis is the C-23 hydroxylation of CT, which is performed by CYP90C1 and CYP90D1.

### YCZ-18 binds to CYP90D1

To characterize the binding target responsible for **YCZ-18** activity in BR biosynthesis inhibition, we cloned the genes of CYP90C1 and CYP90D1 into the pCold-GST expression vector. Using an *E*. *coli* expression system, we attempted to purify these recombinant proteins. Despite substantial efforts to optimize the expression conditions of these genes, we could not obtain the purified recombinant CYP90C1 proteins but had poor expression in soluble fractions. However, we successfully expressed and purified CYP90D1. Thus, we determined the binding affinity of **YCZ-18** to CYP90D1.

**Fig 9:**
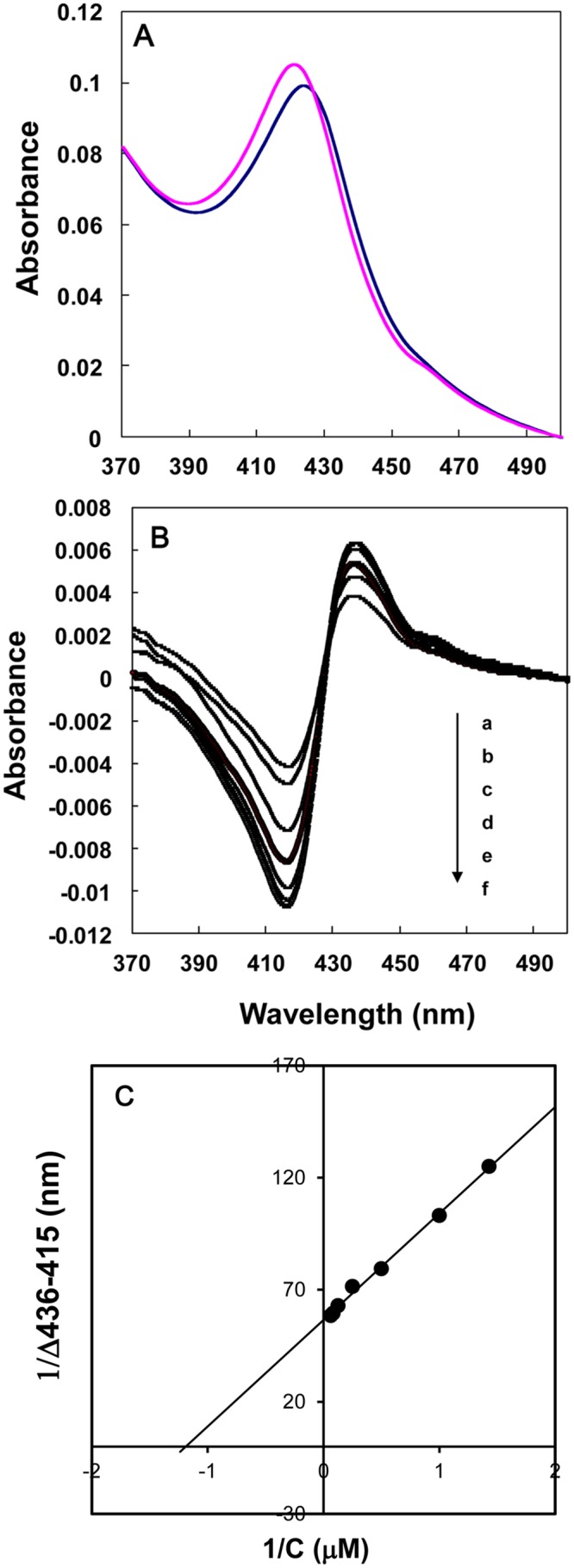
Binding of YCZ-18 to CYP90D1. Absorption spectra of oxidized CYP90D1 (blue line) and its **YCZ-18** complex (pink line). Recombinant CYP90D1 (3.5 μM) was dissolved in 50 mM NaH_2_PO_4_ (pH 7.0) with 0.1% Tween 20 containing 20% glycerol, and **YCZ-18** was added to CYP90D1 at a final concentration of 16 μM (A). Spectrophotometric titration of CYP90D1 with **YCZ-18** induced spectral changes in CYP90D1. **YCZ-18** was added to CYP90D1 (3.5 μM) at various final concentrations (a, 0.7; b, 1; c, 2; d, 4; e, 8; f, 12; g, 16 μM) (B). The spectral dissociation constant was calculated from a double reciprocal plot of absorbance differences, ΔA (436–415 nm) versus the **YCZ-18** concentrations given 0.79 μm (C). The experiment was duplicated to establish reproducibility.

Binding of **YCZ-18** to CYP90D1 was determined by measuring optical difference spectra upon the addition of **YCZ-18** to recombinant CYP90D1. CYP90D1 exhibited a Soret absorption peak at 421 nm, which is characteristic of low-spin P450s (Fig. 9A, pink line). The addition of **YCZ-18** to the CYP90D1 protein induced a type II absorbance shift of the heme Soret band from 421 to 425 nm (Fig. 9A, blue line). This is characteristic of the change from a low to a high spin state of the ferric iron that is usually associated with the direct coordination of the triazole group of the **YCZ-18** to the heme iron of CYP90D1. The dissociation constant K_d_ was determined by titrating the observed spectral absorbance difference (ΔA436-A416) versus the concentration of **YCZ-18** (Fig. 9B). The double reciprocal plot for calculating *K*_*d*_ revealed that the dissociation constant for **YCZ-18** was 0.79 μm (Fig. 9C).

## Discussion

In the present work, we used a variety of methods to investigate the mechanism of **YCZ-18** action and presented evidence that **YCZ-18** is a potent inhibitor of BR biosynthesis with a wide range of applicability to alter the BR levels in *Arabidopsis*. We used three culture methods and demonstrated that **YCZ-18** induced BR-deficient-like dwarf phenotypes in *Arabidopsis* at low doses (Fig. 3–5). Using GC-MS analysis to determine the endogenous levels of **YCZ-18**-treated *Arabidopsis* provided definitive evidence that **YCZ-18** caused the significant decrease in endogenous levels of BRs in *Arabidopsis*. Moreover, we found that the pattern of the endogenous levels of BRs in **YCZ-18**-treated *Arabidopsis* was different from Brz-treated *Arabidopsis*. This observation suggests that **YCZ-18** has a different mode of action from that of Brz. To gain insight into the physiological and cellular mechanisms underlying the reduction in longitudinal growth associated with the **YCZ-18** treatment, we performed a histological analysis. Our transverse stem sections indicated that the **YCZ-18**-induced morphological phenotypes were due to a significant reduction in cell elongation (Fig. 6A, 6B). This result is consistent with the previous observations from BR-deficient mutants and brassinazole-treated *Arabidopsis* [25, 31].

It has been reported that *Arabidopsis* BR biosynthetic mutants exhibit a prolonged vegetative phase and delayed flowering time. For example, the endogenous BR levels in *det2* mutants are less than 10% of that in wild-type plants [31]: the mutants produced more than twice as many rosette leaves as wild-type *Arabidopsis* before flowering. Likewise, *dwf4* also possessed a prolonged vegetative phase and produced approximately twice the number of rosette leaves as the wild type. The flowering time was delayed by approximately 4 days in *dwf4* mutants [45]. These results indicated that altering the endogenous BR levels affected rosette leaf initiation in *Arabidopsis*. Data obtained in the present study indicate that the **YCZ-18**-treated plants produce more than twice as many rosette leaves as the untreated controls (Fig. 5E). Although the molecular mechanism underlying the biological action of **YCZ-18** on promoting rosette leaf growth requires elucidation, our finding suggests that the biological activity of **YCZ-18** on suppressing the *Arabidopsis* growth is effective for over two weeks. Moreover, **YCZ-18** induced a dwarf phenotype of the hypocotyl and produced more rosette leaves in wild-type *Arabidopsis* than in plants without chemical treatment. Indeed, similar dwarf phenotypes could be observed not only in BR-deficient mutants but also in gibberellin (GA)-deficient mutants.

To distinguish the primary site of action of **YCZ-18** between BR biosynthesis and GA biosynthesis, we have previously shown that the dwarf phenotype of *Arabidopsis* seedlings induced with **YCZ-18** in the dark could be rescued by the application of BR but not by GA [29]. Data obtained from the current study provided molecular confirmation through a qPCR analysis of BR-related marker genes. The expression of *TCH4*, which is induced by BR treatment, was downregulated by **YCZ-18** treatment. Further, the expression of *DWF4*, which is a gene sensitively suppressed by BR treatment, was upregulated by **YCZ-18** treatment (Fig. 7A, 7B). Importantly, in the case of the GA-deficient mutant *ga1–5*, the expression levels of *TCH4* and *DWF4* are not altered in comparison with *Arabidopsis* wild-type plants (http://bar.utoronto.ca/efp/cgi-bin/efpWeb.cgi). Thus, although the dwarf phenotype may be due to the deficiency of both BR- and GA-biosynthesis, combining our qPCR data with our previous observations [29], it is clear that **YCZ-18** targets BR biosynthesis.

Another line of evidence indicating that **YCZ-18** is a potent and specific inhibitor of BR biosynthesis is obtained from the qPCR analysis of photosynthesis genes. We found that the expression of the photosynthesis genes *rbcS* and *LHCP* was upregulated by **YCZ-18** in the dark-germinated wild-type *Arabidopsis* plants (Fig. 7C, D). **YCZ-18** also induced promotion of greening in the light-grown plants (Fig. 2 and 3). It has been reported that the BR-deficient mutant *det2* and brassinazole-treated *Arabidopsis* wild-type plants induced expression of photosynthesis genes both at the germination stage in the dark and at the promotion of leaf greening in the light-grown stage. The deficiency of BRs has been considered to be a unique phenomenon that could cause de-etiolation and the induction of photosynthesis gene expression in the dark [23, 31]. In case of *max2*, a F-box deficient mutant that was identified as a stay-green and late senescence mutant *ore9* through mutant screening [46], the hypocotyl elongation of dark-germinated *max2* mutant was found as long as *Arabidopsis* wild-type plant [47]. Similarly, the plant hormone cytokinin was also considered to have activity suppressing leaf senescence, thereby inhibiting hypocotyl elongation in *Arabidopsis* [48]. The mechanism of cytokinin’s inhibition of hypocotyl elongation in *Arabidopsis* is attributed to a secondary effect of ethylene, a plant hormone that promotes senescence, which can be produced by the action of cytokinin [49]. Considering these observations, data obtained in the present work indicate that induction of photosynthesis genes in the dark and the promotion of leaf greening in the light by **YCZ-18** was largely or exclusively due to the primary action of **YCZ-18** on inhibiting BR biosynthesis.

To identify the target of **YCZ-18** in BR biosynthesis, we conducted a feeding experiment involving the application of BR biosynthesis intermediates to **YCZ-18-**treated *Arabidopsis*, followed by the determination of the binding affinity of **YCZ-18** to purified recombinant enzymes of interest. Feeding of cathasterone and teasterone to **YCZ-18**-treated *Arabidopsis* demonstrated that the probable target site for **YCZ-18** in BR biosynthesis was the C-23 hydroxylation of cathasterone (Fig. 8). Binding studies of **YCZ-18** to CYP90D1 provided evidence indicating that **YCZ-18** induced a typical type II binding spectrum with a dissociation constant of approximately 0.79 μM (Fig. 9). Genetic analysis of the C-23 hydroxylase mutants in BR biosynthesis indicated that CYP90C1 and CYP90D1 were two closely related genes with redundant functions as C-23 hydroxylases in BR biosynthesis [19, 50]. The disruption of CYP90C1 in the *rot3* mutants results in a weak dwarf phenotype [51] but causes no appreciable alteration of the endogenous BR levels [50]. Further, CYP90D1 deficiency does not show any visible changes in *Arabidopsis* morphology [50]. In contrast, the double mutant for these P450 enzymes exhibits a severe dwarf phenotype [19, 50]. Because the soluble recombinant protein of CYP90C1 was poorly expressed in *E*. *coli*, the binding analysis for **YCZ-18** and CYP90C1 could not be performed. However, based on the observations of the **YCZ-18**-induced morphological changes in *Arabidopsis* seedlings, we anticipate that **YCZ-18** blocks C-23 hydroxylation of BR biosynthesis, thereby interfering with both CYP90C1 and CYP90D1 (Fig. 1) because **YCZ-18** can induce severe BR-deficient-like phenotypes in *Arabidopsis* at low doses (Fig. 3–6).

Data obtained from binding analysis combined with the quantitative analysis gave definitive evidences that **YCZ-18** is a new BR biosynthesis inhibitor. Binding analysis indicated that one of the primary site of action of **YCZ-18** is the C23 hydroxylase. Additionally, the profiles of BR intermediates in **YCZ-18** treated Arabidopsis is quite different from Brz treated plants (Table 1), Interestingly, we found the levels of 3-dehydro-6-deoxoteasterone in **YCZ-18** treated Arabidopsis is almost in the same levels as that of control (Table 1), implying that **YCZ-18** may also interfering the steps between 3-dehydro-6-deoxoteasterone and 6-deoxocastasterone.

Brassinazole has been identified as the first synthetic small-molecule compound targeting C-22 hydroxylase (DWF4) in BR biosynthesis (Fig. 1) [26]. Data obtained in this work provide evidence for the first time that **YCZ-18** targets the C-23 hydroxylation of cathasterone in BR biosynthesis, causing remarkable effects on plant growth and development. Therefore, it is worthwhile to emphasize that the most important finding in this work is that the step of C-23 hydroxylation of BRs is an appropriately sensitive target for inhibitors. Moreover, because the **YCZ-18** targeting step is different from brassinazole in BR biosynthesis, **YCZ-18** is a new important molecular tool for elucidating the functions of BRs.

Until now, brassinazole has been widely used for BR research, both for the elucidation of BR functions in plant physiology and for the identification of BR signal transduction components [52]. The use of chemical inhibitors in genetic screens provides an efficient way to identify novel mutants, which has emerged as a useful strategy to study biological systems in plants [14]. In the last century, mutagenesis strategies have played a central role in elucidating biological processes by investigating the relationships between genes and phenotypes, called classical genetics. Genetic approaches cause permanent, irreversible changes in the genetic and phenotypic make-up. The problems associated with classical genetics include genetic lethality, redundancy and tissue/development-specific expression. Because small molecules target proteins by modulating their functions, they can overcome the problems associated with classical genetics. Another advantage for small molecules over classical genetics is that they are easy to apply to different plant species and different stages of plant growth and development. In some cases, small molecules also provide selective inhibition of certain isoforms of an enzyme. For example, several small-molecule inhibitors of phosphoinositide 3-kinases were recently profiled. Their ability to inhibit specific kinase isoforms is useful for elucidating the respective roles of these isoforms in insulin signaling [53]. Small molecules are especially useful as general tools to elucidate various biological processes. As in the present work, **YCZ-18** exhibits potent biological activity upon inducing BR-deficient-like phenotypes in *Arabidopsis*. In addition, **YCZ-18** and brassinozole target different enzymes. Therefore, further experiments with **YCZ-18** to explore the biological processes related to BRs may provide new insights into the detailed mechanism of BR biosynthesis and its regulation.

## Conclusions

We present three independent lines of evidence indicating that **YCZ-18** is a potent inhibitor of BR biosynthesis with a wide range of applicability for altering BR levels in *Arabidopsis*. (1) Under three culture methods, **YCZ-18** induces an *Arabidopsis* phenotype that is similar to that of BR-deficient mutants. (2) **YCZ-18** regulates the expression of BR-responsive genes, which was shown in both BR-deficient mutants and brassinazole-treated plants. (3) Quantitative analysis on BR levels in **YCZ-18** treated *Arabidopsis* indicated that **YCZ-18** significantly decreased the endogenous levels of BRs. (4) **YCZ-18** shows high binding affinity to recombinant CYP90D1 protein, suggesting that one of the primary sites of action of **YCZ-18** is the C-23 hydroxylation of the side chain of BRs. Combining these results with our previously reported observations [28], we conclude that **YCZ-18** is a potent BR biosynthesis inhibitor and has a new target site, C23-hydroxylation in BR biosynthesis.
